# Modeling of the General Trends of Reactivity and Regioselectivity in Cyclopentadiene–Nitroalkene Diels–Alder Reactions

**DOI:** 10.3390/molecules30112467

**Published:** 2025-06-04

**Authors:** Adrianna Fałowska, Stanisław Grzybowski, Daniel Kapuściński, Karol Sambora, Agnieszka Łapczuk

**Affiliations:** Department of Organic Chemistry and Technology, Cracow University of Technology, Warszawska 24, 31-155 Krakow, Polanddaniel.kapuscinski@student.pk.edu.pl (D.K.); karol.sambora@student.pk.edu.pl (K.S.)

**Keywords:** reactivity, (4+2) cycloaddition, cyclic dienes, conjugated nitroalkenes, CDFT, MEDT, ELF, cyclopentadiene, Diels–Alder reaction

## Abstract

This study presents a theoretical investigation of the electronic properties of mono- and pentasubstituted cyclopentadiene analogs and variously substituted conjugated nitroalkenes bearing electron-donating and electron-withdrawing groups. Conceptual Density Functional Theory (CDFT) and Electron Localization Function (ELF) analyses were employed to characterize the global and local reactivity indices of the reactants. The obtained data provided insights into the nucleophilic and electrophilic nature of the investigated systems, allowing for the prediction of their reactivity patterns in Diels–Alder reactions. A reactivity model for conjugated alkenes toward cyclopentadienes was developed based on correlation analysis using Hammett substituent constants. This approach enabled the prediction of reaction polarity in (4+2) cycloaddition processes, providing insight into how the electronic effects of substituents influence the reaction course. These findings contribute to a deeper understanding of structure–reactivity relationships in Diels–Alder processes.

## 1. Introduction

**Scheme 1:**
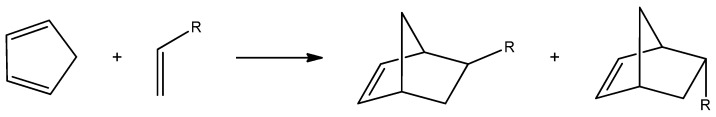
The general scheme of the Diels–Alder reaction between cyclopentadiene and a simple alkene.

Norbornenes are cyclic hydrocarbons that contain a strained double bond in their structure, which is responsible for their high reactivity [1,2]. They have numerous applications as monomers and intermediates in organic synthesis, including in medicine as building blocks for the production of pharmaceuticals [3]. Norbornenes also participate in various polymerization reactions. One of the most commonly employed methods for converting norbornenes into polymers is ring-opening metathesis polymerization (ROMP) [4]. These compounds can be synthesized via the Diels–Alder (DA) reaction between cyclopentadiene and an alkene (Scheme 1). The DA reaction is one of the most well-known cycloaddition processes, used to construct cyclic compounds through the reaction of a conjugated diene with a dienophile [5,6,7]. First described in 1928, this reaction has found countless applications [8]. It is a key strategy for generating complex molecular architectures with excellent atom economy and minimal waste. Undoubtedly, the DA reaction is among the most powerful tools in organic synthesis [9].

DA cycloaddition between cyclopentadiene and variously substituted alkenes leads to the formation of norbornene and its derivatives. Due to the stereo orientation of substituents derived from the alkene structure, the products of this reaction typically exist as two stereoisomers characterized by *endo* and *exo* orientation [5,10,11].

**Scheme 2:**
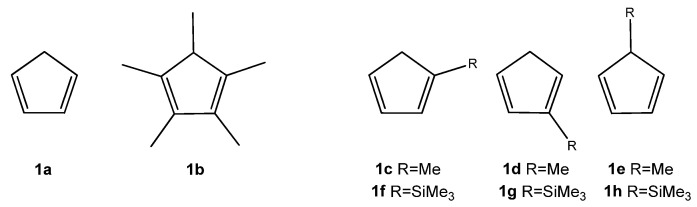
Structures of tested analogs of cyclopentadiene (**1a–h**).

**Scheme 3:**
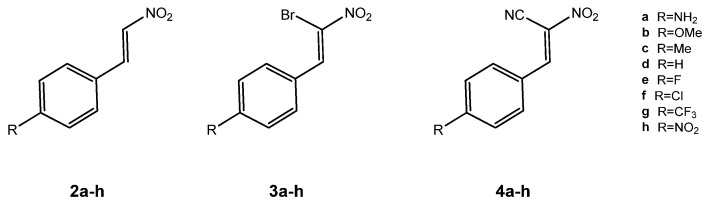
Structures of tested para-substituent analogs of conjugated nitroalkenes (**2–4**).

While the DA reactions between the parent cyclopentadiene and nitroalkenes have been described in the literature [12,13], studies involving substituted analogs of cyclopentadiene in these transformations remain scarce. Therefore, our objective was to compare the reactivity of unsubstituted cyclopentadiene (**1a**) with its mono- and pentasubstituted analogs (**1b**–**h**) (Scheme 2). For this purpose, we assembled a series of conjugated nitroalkenes (**2a**–**h**) bearing either electron-releasing (ER) or electron-withdrawing (EW) substituents on the phenyl ring. Additionally, we expanded the study by including two classes of 1-phenyl-2-nitroethenes analogs: compounds substituted with a bromine atom (**3a**–**h**) and those bearing a cyano group at the α-carbon position (**4a**–**h**) (Scheme 3). Cyclopentadiene (**1a**) and methylcyclopentadiene (**1c**–**e**) are commonly produced through the thermal cracking of their respective commercially available dimers. 1,2,3,4,5-Pentamethylcyclopentadiene (**1b**) is synthesized through a multi-step process using diethyl ketone as the starting material [4]. Trimethylsilylcyclopentadienes (**1f**–**h**) were obtained by reacting sodium cyclopentadienide with trimethylsilyl chloride [5]. Both methylcyclopentadiene and trimethylsilylcyclopentadiene exhibit isomerism. Substituted (*E*)-1-phenyl-2-nitroethenes (**2a**–**h**) were obtained via a Henry reaction. Substituted (*Z*)-1-bromo-1-nitro-2-phenylethenes (**3a**–**h**) were synthesized through bromination of (*E*)-1-phenyl-2-nitroethenes, followed by dehydrobromination [14]. In contrast, (2*E*)-3-phenyl-2-nitroprop-2-enenitriles (**4a**–**h**) can be prepared via a condensation reaction between the appropriate aldehyde and nitroacetonitrile [15].

We conducted a topological analysis of the electronic structure of cyclopentadienes (**Cp**) and conjugated nitroalkenes (**CNA**). Additionally, we examined reactivity indices at the ground state of the reagents to predict their reactivity and regioselectivity in bimolecular reaction systems, particularly in DA reactions [16,17,18]. We also proposed a reactivity model based on a correlation analysis of selected electronic parameters. Recent theoretical studies have established that a significant number of DA reactions proceed via a polar mechanism, in which the reactivity is governed by nucleophilic/electrophilic interactions at the transition state structures (TSs) [19]. Within this framework, the global electron density transfer (GEDT) occurring at TSs has been identified as a key descriptor of the polar character and feasibility of DA reactions. Therefore, the analysis of global reactivity indices—namely electrophilicity (ω) and nucleophilicity (N)—at the ground state of the reactants provides valuable insight into the driving forces behind such polar processes. Moreover, when non-symmetric reagents are involved, two regioisomeric cycloadducts can be formed. In polar DA (P-DA) reactions, regioselectivity is primarily dictated by the most favorable two-center interaction between the most nucleophilic and the most electrophilic atomic centers of the reacting species. The local reactivity descriptors derived from the Parr functions (Pₖ⁺ and Pₖ⁻) enable the identification of these key reactive sites and allow for reliable prediction of regioselectivity. In this study, both global and local DFT-based reactivity indices are employed to rationalize the observed selectivity and polarity of the cycloaddition reactions under investigation.

## 2. Results and Discussion

To gain deeper insight into the structure and properties of both substrates **Cp** (**1a**–**h**) and **CNA** (**2a**–**h**, **3a**–**h**, **4a**–**h**), we performed an Electron Localization Function (ELF) and Conceptual DFT (CDFT) analysis. The results of these analyses were then interpreted to propose the most probable course of cyclopentadienes in the DA reaction between **Cp** and **CNA**.

### 2.1. The Electron Localization Function (ELF)

**Figure 1:**
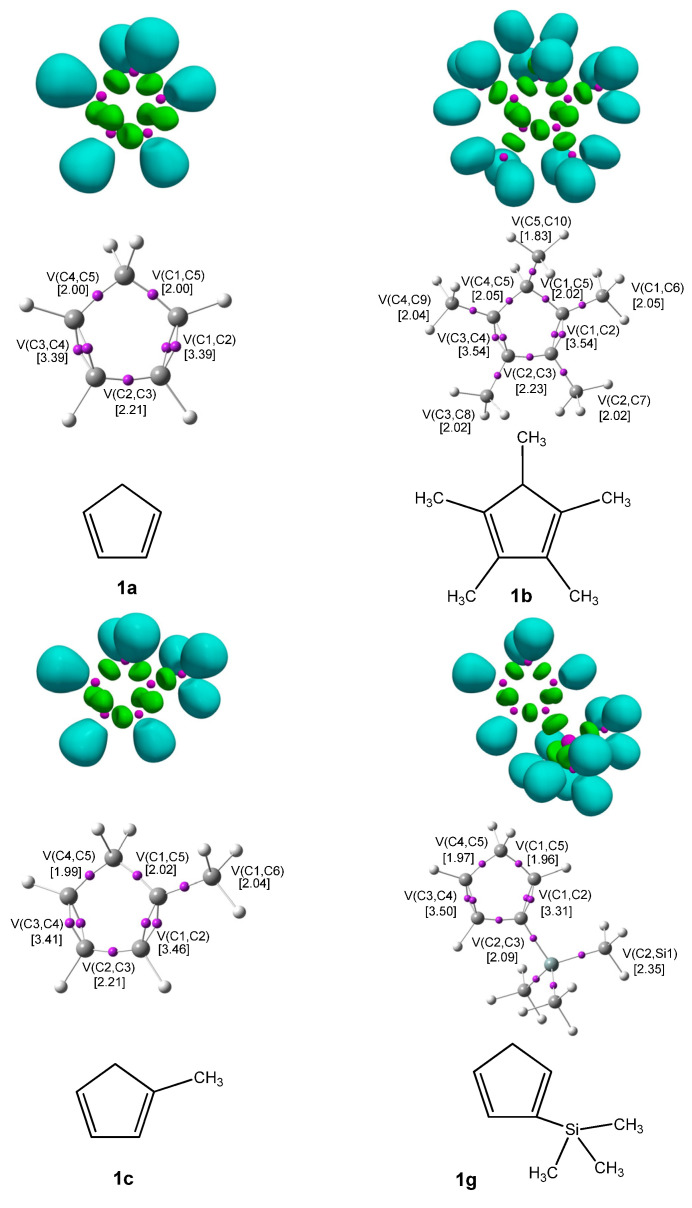
ωB97X-D/6-311G(d): ELF localization domains of **Cp** represented at an isosurface value of ELF = 0.75 on the top side. ELF basin attractor positions, together with the most representative valence basin populations, in the center and the proposed ELF-based Lewis structures.

**Figure 2:**
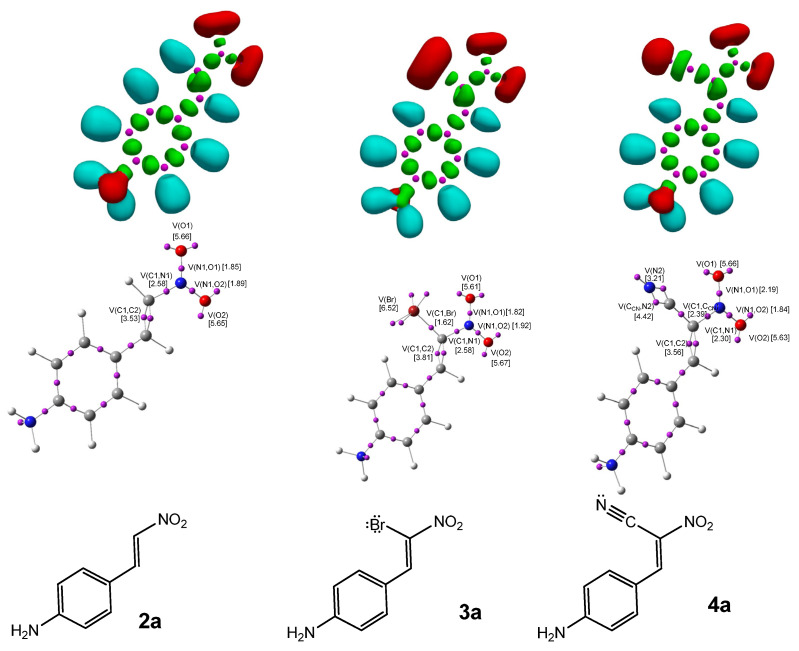
ωB97X-D/6-311G(d): ELF localization domains of **CNA (2**–**4a**) represented at an isosurface value of ELF = 0.75 on the top side. ELF basin attractor positions, together with the most representative valence basin populations, in the center and the proposed ELF-based Lewis structures.

The ELF [20] serves as a powerful method for analyzing the electronic structure of organic molecules, offering both qualitative and quantitative insights. Therefore, based on the known structure–reactivity relationships of **CNA**, a topological ELF analysis was carried out for **CNA** (**2a**–**h**, **3a**–**h**, **4a**–**h**) and **Cp** (**1a**–**h**) to examine their electronic structures and gain insights into their potential reactivity in DA reactions. Figure 1 displays the ELF localization domains, the ELF basin attractor positions, and corresponding proposed Lewis-like structures for **Cp 1**, while Figure 2 shows the same data for **CNA**. The most relevant valence basin populations are summarized in Table 1 and Table 2.

The topological ELF analysis of **Cp** (**1a–f**) reveals the presence of two disynaptic basins in the C1–C2 and C3–C4 bonding regions, denoted as V(C–C) and V′(C–C), with a total electron population ranging from 3.24 to 3.54 e, depending on the substituents present on the cyclopentadienyl ring. In symmetric compounds, these values are equal. These results suggest a double bond character between the C1–C2 and C3–C4 carbon atoms. The remaining bonds in the ring correspond to single V(C–C) bonds with populations ranging from 1.99 to 2.24 e. The C–R bonding region (where the substituent is attached) is characterized by the presence of V(C, R) disynaptic basins, with populations ranging from 2.02 to 2.44 e when the substituents are in positions 1 or 2, and slightly lower values of 1.81 to 1.89 e when the substituent is attached to the C5 atom of the ring. These values indicate that the C–R bond exhibits a single bond character.

The ELF topological analysis of the most relevant fragment in the **CNA** (Figure 2) reveals the presence of two pairs of disynaptic basins, V(C1,C2) and V′(C1,C2), with a combined electron population of approximately 3.5 e, depending on the substituent R in the phenyl ring (Table 2). This indicates a double bond character between C1 and C2. The nitro group is bonded to C1 through a single bond, with a total electron population of 2.31–2.32 e for compounds **2b**–**h**, and a slightly higher population of 2.58 e for **2a**.

Both oxygen atoms in the nitro group are connected to the nitrogen atom via single bonds, with electron populations ranging from 1.84 to 1.89 e. The analysis also shows the presence of two monosynaptic basins, V(O1) and V′(O1), located at the O1 atom, integrating a total electron population of 5.62–5.67 e, and one monosynaptic basin, V(O2), at the O2 atom, with a population of 2.82–2.90 e. Compounds **2b**–**h** exhibit two monosynaptic basins at the N1 atom, V(N1) and V′(N1), with a combined electron population of 0.30–0.32 e. In contrast, compound **2a** shows no monosynaptic basin associated with the N1 atom in the ELF analysis.

In **CNA** (**3a**–**h**) (Table 3), similarly to the previous cases, a double bond is observed between the C1 and C2 atoms. A disynaptic basin V(C1, Br) with an electron population of approximately 1.6e can be observed. On the bromine atom, monosynaptic basins with a total electron population of 6.5e are noted. In the case of conjugated nitroalkenes (**4a–h**), the topological analysis of the ELF revealed two disynaptic basins, V(C1,C2) and V′(C1,C2), with a combined electron population of approximately 3.5 e, characteristic of a localized C=C double bond. The nitro group is connected to the carbon framework via a single bond, represented by a disynaptic basin V(C1,N1) with an electron population ranging from 2.25 to 2.30 electrons, indicating a typical single bond character. The presence of disynaptic basins V(N1,O1) and V(N1,O2), each integrating between 1.84 and 2.24 electrons, confirms the single bond nature of the N–O interactions. Each oxygen atom (O1 and O2) exhibits two monosynaptic basins, V(O) and V′(O), with a combined electron population of approximately 5.63 electrons per atom. This electronic distribution reflects the presence of three lone pairs on each oxygen atom, consistent with their high electronegativity and the resonance delocalization characteristic of the nitro group.

Within the case of **CNA** (**4a**–**h**) (Table 4), the topological analysis of the ELF revealed two disynaptic basins, V(C1,C2) and V′(C1,C2), with a combined electron population of approximately 3.5 e, characteristic of a localized C=C double bond. A disynaptic basin V(C1,C_CN_) with an electron population of 2.39 e was also detected between the C1 carbon atom and the nitrile substituent, corresponding to a single C–C bond. Furthermore, two additional disynaptic basins were observed, integrating 4.43 e and 4.54 e, respectively, suggesting a highly polarized double bond, possibly influenced by conjugation EW groups. A monosynaptic basin V(N2), located at the nitrogen atom of the nitro group, was identified with an electron population ranging from 3.16 e to 3.21 e, indicative of significant lone pair localization. The structure of the nitro group is consistent with the characteristics observed in the previous compounds.

The Lewis structures of the investigated compounds were elucidated based on the results of ELF topological analysis (Figure 2).

### 2.2. CDFT

#### 2.2.1. Global Electronic Properties

As a first step, the electronic properties of the investigated cyclopentadienes were determined. Based on their optimized geometries, we calculated the chemical hardness (η) and chemical potential (μ), followed by the global electrophilicity index (ω) [21]. The calculations were performed using the ωB97X−D/6-311G(d) theory level in the gas phase. 

All of the analyzed **Cp** exhibited strong or even superstrong nucleophilicity, with nucleophilicity N index [22] values ranging from 3.32 eV for monosubstituted derivatives to 4.14 eV for the pentasubstituted cyclopentadiene. The presence of methyl substituents increases the global nucleophilicity of the cyclopentadiene analogs; however, their position within the ring has only a minor influence. A similar effect is observed for the trimethylsilyl group. At the same time, their global electrophilicity ω index values were relatively low, allowing them to be classified as moderate or marginal electrophiles [23]. All values are summarized in Table 5.

In the next step, we investigated the electronic properties of the **CNA**. Table 6 summarizes the relationship between the electrophilicity values and the Hammett sigma constants. In the literature, it was found that many examples of the application of these constants for the interpretation of the organic reactivity were described [24,25,26]. In line with our initial assumptions, all the studied nitroalkenes exhibit high electrophilic character and can be classified as strong electrophiles. The presence of EW groups on the phenyl ring leads to an increase in the global electrophilicity index while simultaneously reducing the global nucleophilicity index. In contrast, strong ER substituents enhance the nucleophilicity, making the corresponding compounds both strong nucleophiles and strong electrophiles. Substitution at the C1 position of cyclopentadiene with a bromine atom results in an increase in global electrophilicity; this effect is even more pronounced with cyano substitution. Overall, compounds **4a**–**h** exhibit higher electrophilicity than their unsubstituted analogs.

#### 2.2.2. Local Electronic Properties

**Figure 3:**
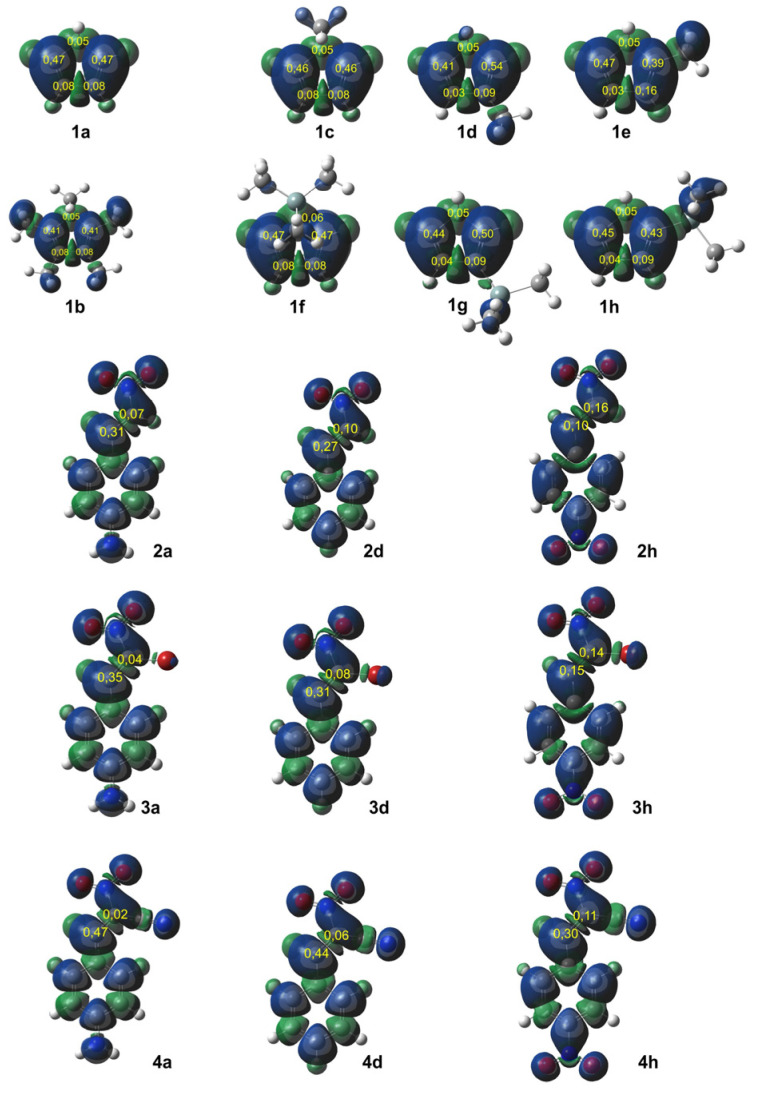
Local electron properties of **Cp** (**1a**–**h**) and **CNA** presented in three-dimensional visualization of Mulliken‘s atomic spin densities (isovalue = 0.0004) with nucleophilic functions of P_k_^-^ for substrates (**1**) and electrophilic functions of P_k_^+^ for substrates (**2**–**4**) given in yellow.

**Scheme 4:**
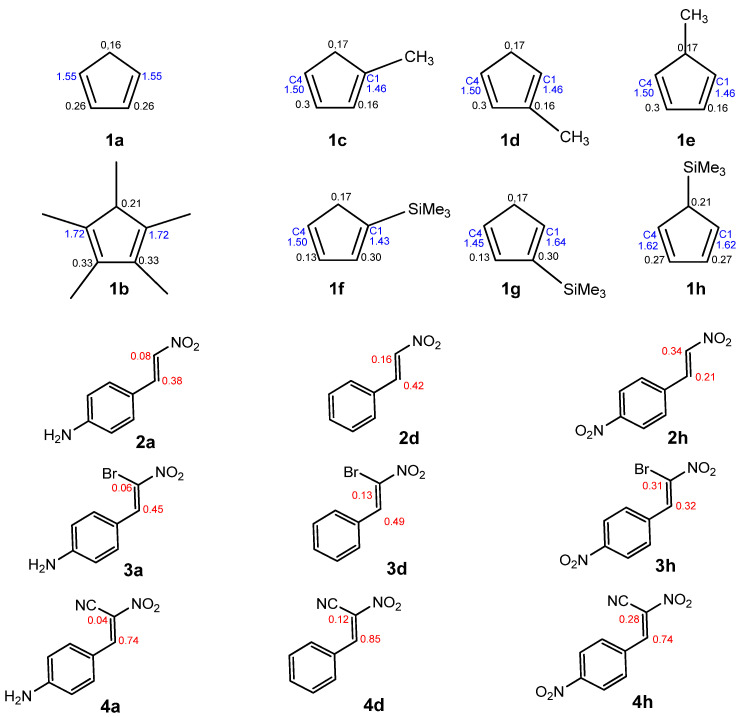
The local nucleophilicity N_k_ indexes of the carbon atoms for the **Cp** (**1a**–**h**) substrates are given in blue. The local electrophilicity ω_k_ indexes for the **CNA** (**2**–**4**) substrates are given in red. The values are expressed in the eV unit.

In DA reactions between **Cp** and **CNA**, regioisomers could hypothetically form. To investigate the reaction selectivity and preferred direction, we analyzed both global and local electronic properties. All numerical data were compiled in Table 7 and Table 8. However, visualizations were provided only for selected compounds (Figure 3, Scheme 4). To identify the most nucleophilic and electrophilic sites of the species involved in these reactions, we analyzed the electrophilic P_k_+ and nucleophilic P_k_⁻ Parr functions, along with the local electrophilicity ω_k_ and local nucleophilicity N_k_ [27]. This method provided insights into the most electrophilic and nucleophilic regions of the molecules. **Cp** compounds are classified as strong nucleophiles. In the case of symmetric compounds (**1a, b, c, f**), the carbon atoms at positions C1 and C4 exhibit identical local nucleophilicity. For monosubstituted **Cp** compounds at position C1 (**1d, h**), the most nucleophilic atom is C4, whereas in cyclopentadiene analogs with a substituent at position C2 (**1d, g**), the highest local nucleophilicity is observed at C1.

All conjugated nitroalkenes were classified as strong electrophiles. In most cases, the electrophilic center is located at carbon C2. Exceptions include compounds **2h** and **4h**, where the nitro group is in the para-position of the phenyl group. In these cases, both carbon atoms in the conjugated nitroalkenes exhibit similar electrophilicity (**3h**), or slightly higher electrophilicity is observed at C1 (**2h**).

### 2.3. Reactivity of Cp toward CNA

**Scheme 5:**
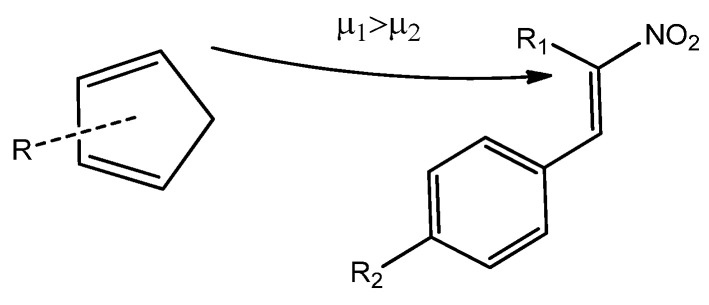
Visualization of electron density flux in the course of the DA reactions of cyclopentadienes (**1**) with conjugated nitroalkenes (**2**–**4**).

Based on the data gathered so far, we can now examine the reactivity of **Cp** toward **CNA** in DA reactions. The chemical potential is a useful parameter for determining the direction of electron density flow between the reactants [28]. The chemical potential of **CNA** (**2**–**4**) ranges from −5.79 to −4.25 eV, while for **Cp** (**1a**–**h**), this value is consistently higher in each case (Table 5). Based on these values, we can conclude that the electron density will flow from the cyclopentadiene to the conjugated nitroalkenes, indicating a forward electron density flux (FEDF) [29] from the diene to the dienophile (Scheme 5).

Determining which reactant acts as the electrophile and which as the nucleophile—along with identifying the most electrophilic and nucleophilic centers—is essential in reactions that can yield multiple isomeric products [30,31]. In addition, a thorough analysis of the local electronic characteristics of the atoms involved in bond formation is necessary to theoretically predict the most favorable reaction pathway during the formation of the new ring system [32].

Due to the higher local electrophilicity of the β-carbon atom (C2, adjacent to the phenyl group in **CNA**), certain reaction pathways of **CNA** (**2a**–**g**, **3a**–**g**, **4a**–**g**) with unsymmetrical **Cp** are expected to be favored. However, in the case of conjugated nitroalkenes substituted with a nitro group in a phenyl ring (**2–4h**), the reaction selectivity shifts, as the C1 carbon atom becomes the most electrophilic center in these derivatives.

**Scheme 6:**
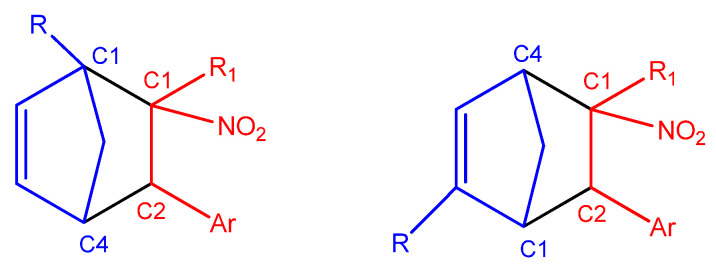
Most probable regioisomeric products of the DA reactions between substituted **Cp** (**1c,d,f,g**) and **CNA** (**2**–**4 a**–**g**), based on local reactivity indices.

In the case of asymmetrically substituted **Cp**, the nucleophilicity of the carbon atoms is strongly influenced by the position of the substituent. For 1-substituted cyclopentadienyl derivatives, the more nucleophilic carbon C4 preferentially forms a bond with the more electrophilic β-carbon (C2) of the conjugated alkenes (**2a**–**g**, **3a**–**g**, **4a**–**g**) during the DA reaction. In 2-substituted systems, the C1 carbon exhibits the highest nucleophilicity and is, therefore, most likely to engage in bond formation with the β-carbon (C2) of the nitroalkene. For **CNA 2–4h**, the presence of EW substituents at the α-position may lead to the formation of alternative regioisomeric products (Scheme 6).

By analyzing the difference in global electrophilicity (Δω) between the components involved in the cycloaddition reaction, it is possible to assess the polar character of the process. Cycloadditions between strong electrophiles and strong nucleophiles are expected to proceed via a polar mechanism when Δω > 1 eV. In contrast, when Δω < 1 eV, the reaction is predicted to follow a non-polar pathway.

A comparative analysis of the global electrophilicity indices of cyclopentadienes and conjugated nitroalkenes reveals that the presence of ER substituents on the phenyl ring of conjugated nitroalkenes favors a polar DA reaction. Moreover, for cyano-substituted conjugated nitroalkenes, the reaction is polar regardless of the nature of the substituent.

The global electrophilicity differences (Δω) for all investigated DA reactions were computed and are presented in the Supplementary Materials.

**Figure 4:**
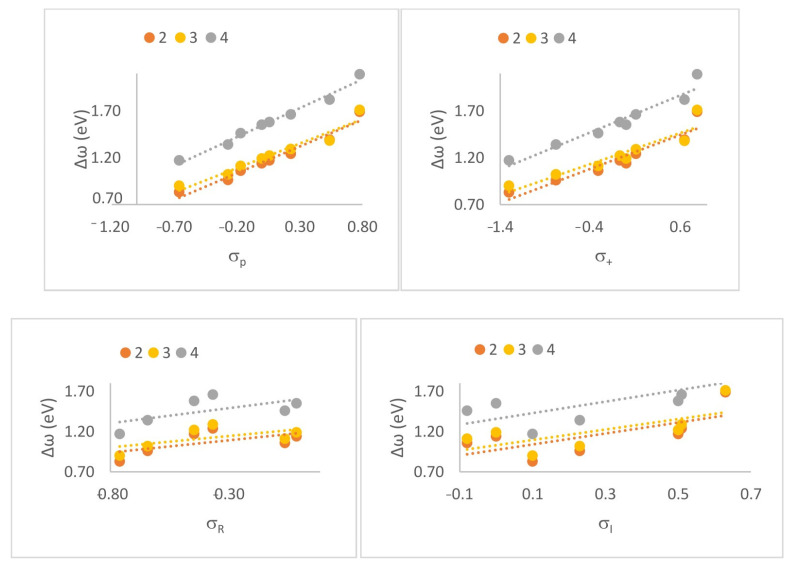
Linear correlation between the global electrophilicity difference (Δω) and Hammett σₚ constants for CNA derivatives in DA reactions with substituted cyclopentadienes.

To better understand the role of substituents in modulating the polarity and reactivity of the studied Diels–Alder reactions, we performed a classical Hammett-type correlation analysis. While global and local DFT-based reactivity descriptors provide mechanistic insight into the electronic properties of the reacting species, they do not directly quantify the influence of individual substituents in a chemically intuitive way. In contrast, Hammett σ constants are well-established empirical parameters that capture the electron-donating or electron-withdrawing nature of substituents through resonance and inductive effects. While not essential to understanding the mechanism per se, this correlation provides a valuable bridge between classical physical organic chemistry and modern theoretical approaches. By correlating the calculated reaction polarity index (Δω) with the Hammett parameters (σ⁺, σₚ, σᴿ, σᴵ) [16], we aimed to establish a quantitative model linking substituent electronic properties to the global polarity of the reaction (Figure 4). Among the tested constants, σ_p_ showed the strongest linear correlation (R = 0.991 for the **4+Cp** reaction, R = 0.979 for compound 2 with **Cp**, and R = 0.970 for compound 3 with **Cp** (Table 9)), suggesting that both inductive and resonance effects are transmitted from the substituents to the reaction center and influence the reaction’s polar character.

## 3. Experimental

### Computational Details

Density functional theory (DFT) calculations were performed using the hybrid ωB97X−D functional [17], which includes long-range exchange corrections (denoted by X) and semiclassical London dispersion corrections (indicated by the −D suffix). The standard 6−311G(d) basis set [18] incorporating d-type polarization functions for second-row elements was employed throughout. Geometry optimizations were carried out using the Berny algorithm [33,34]. This computational approach is well-established for studying the mechanistic pathways of cycloaddition reactions [35,36,37,38]. 

The topological analyses of the ELF [20,28,39] were performed with TopMod 09 [40] software based on monodeterminantal wavefunctions over a grid with a spacing of 0.1 atomic units.

- The essential parameters for the reactivity descriptors were computed using the following formula:
- The chemical potential [21,28,41,42,43]: µ ≈ (E_HOMO_ + E_LUMO_)/2
- The chemical hardness [41,43,44]: η ≈ (E_LUMO_ − E_HOMO_)
- The global electrophilicity index [28]: ω = (µ^2^/2η)
- The global nucleophilicity index [22]: N = E_HOMO_(Nu) − E_HOMO_(TCE), where tetracyanoethylene (TCE) is the reference
- The local electrophilicity was calculated based on global properties and the Parr function (P^+^_k_ or P^−^_k_) [27]: ω_k_ = P^+^_k_·ω
- The local nucleophilicity: N_k_ = P^−^_k_·N

## 4. Conclusions

A comprehensive ELF topological analysis was performed for both cyclopentadienes and conjugated nitroalkenes, complemented by global and local reactivity descriptors obtained via CDFT. Based on this analysis, cyclopentadienes were classified as strong nucleophiles, while conjugated nitroalkenes were identified as strong electrophiles. The DA reactions between **Cp** and **CNA** are predicted to proceed through a polar mechanism when EW substituents are located at the para-position of the phenyl ring in the conjugated nitroalkenes component. Interestingly, in the case of CN-substituted **CNA**, even compounds bearing ER substituents undergo polar DA reactions.

Local electrophilicity and nucleophilicity indices indicate that the β-carbon atom (C2) is the most electrophilic center in **CNA 2a**–**g**, **3a**–**g**, **4a**–**g**, whereas for **CNA** substituted with an additional nitro group (**2**–**4h**), the α-carbon (C1) becomes the most electrophilic site. These findings demonstrate that the course of the cycloaddition reaction can be modulated by the electronic nature and position of the substituents in the reacting components.

A reactivity model for conjugated alkenes toward cyclopentadienes was developed based on correlation analysis using Hammett substituent constants. This approach enabled the prediction of reaction polarity in DA processes, providing insight into how electronic effects of substituents influence the reaction course.

In general, the obtained results offer a general conclusion regarding the wide range of the nitrosubstituted components of the Diels–Alder processes. 

## Figures and Tables

**Table 1:** ωB97X-D/6-311G(d): the most significant ELF valence basin populations N for molecules **Cp 1a**–**h**, given as average number of electrons [e].

	1a	1b	1c	1d	1e	1f	1g	1h
ELF basins	N [e]	N [e]	N [e]	N [e]	N [e]	N [e]	N [e]	N [e]
V(C1,C2)	1.74	1.76	1.69	1.78	1.67	1.57	1.6	1.74
V′(C1,C2)	1.65	1.78	1.77	1.67	1.71	1.67	1.71	1.68
V(C2,C3)	2.21	2.23	2.21	2.22	2.19	2.19	2.09	2.24
V(C3,C4)	1.74	1.76	1.74	1.75	1.71	1.69	1.71	1.68
V′(C3,C4)	1.65	1.78	1.67	1.65	1.67	1.76	1.79	1.74
V(C4,C5)	2.00	2.05	1.99	2.00	2.02	1.98	1.97	2.00
V(C1,C5)	2.00	2.00	2.02	2.01	2.02	1.90	1.96	2.01
V(C1,R1)		2.05	2.04			2.44		
V(C2,R2)		2.02		2.02			2.35	
V(C3,R3)		2.02						
V(C4,R4)		2.04						
V(C5,R5)		1.83			1.81			1.89

**Table 2:** ωB97X-D/6-311G(d): the most significant ELF valence basin populations N for molecules **CNA 2a–h**, given as average number of electrons [e].

	2a	2b	2c	2d	2e	2f	2g	2h
ELF basins	N [e]	N [e]	N [e]	N [e]	N [e]	N [e]	N [e]	N [e]
V(C1,C2)	1.78	1.74	1.79	1.76	1.76	1.76	1.76	1.75
V′(C1,C2)	1.75	1.78	1.72	1.76	1.76	1.76	1.75	1.75
V(C1,N1)	2.58	2.32	2.32	2.32	2.32	2.32	2.32	2.31
V(N1,O1)	1.85	1.84	1.85	1.84	1.85	1.84	1.85	1.84
V′(N1,O2)	1.89	1.87	1.86	1.87	1.89	1.87	1.87	1.88
V(O2)	2.81	2.82	2.80	2.80	2.80	2.80	2.82	2.80
V(O2)	2.85	2.85	2.85	2.85	2.85	2.85	2.85	2.81
V(N1)		0.15	0.15	0.15	0.15	0.16	0.16	0.16
V′(N1)		0.15	0.15	0.15	0.15	0.16	0.16	0.16
V(O1)	2.83	2.8	2.83	2.82	2.81	2.82	2.85	2.85
V′(O1)	2.82	2.86	2.81	2.82	2.81	2.8	2.8	2.82
V(C6,R)	2.26	1.57	2.01		1.06	1.49	2.24	2.3

**Table 3:** ωB97X-D/6-311G(d): the most significant ELF valence basin populations N for molecules **CNA 3a–h**, given as average number of electrons [e].

	3a	3b	3c	3d	3e	3f	3g	3h
ELF basins	N [e]	N [e]	N [e]	N [e]	N [e]	N [e]	N [e]	N [e]
V(C1,C2)	1.86	1.85	1.94	1.89	1.89	1.89	1.94	1.90
V′(C1,C2)	1.95	1.94	1.84	1.89	1.89	1.89	1.82	1.86
V(C1,Br)	1.62	1.63	1.64	1.64	1.64	1.65	1.67	1.67
V(Br)	1.95	2.00	2.70	1.96	1.97	1.96	2.69	1.97
V′(Br)	2.02	1.96	2.00	1.96	1.97	1.96	1.82	1.90
V′(Br)	1.32	1.33	1.85	1.31	1.31	1.31	2.02	1.36
V′(Br)	1.26	1.27		1.31	1.31	1.31		1.29
V(C1,N1)	2.58	2.28	2.27	2.26	2.27	2.27	2.26	2.27
V(N1,O1)	1.82	1.82	1.83	1.83	1.83	1.82	1.84	1.83
V′(N1,O2)	1.92	1.90	1.90	1.91	1.91	1.91	1.91	1.90
V(O2)	2.86	2.78	2.81	2.81	2.82	2.79	2.81	2.81
V′(O2)	2.81	2.89	2.85	2.84	2.84	2.85	2.84	2.85
V(N1)		0.17	0.18	0.17	0.17	0.18	0.18	0.18
V′(N1)		0.17	0.17	0.17	0.17	0.18	0.19	0.18
V(O1)	2.82	2.80	2.79	2.82	2.82	2.77	2.80	2.81
V′(O1)	2.79	2.80	2.81	2.77	2.78	2.81	2.77	2.78
V(C6,R)	2.31		2.01		1.07	1.50	2.24	2.31

**Table 4:** ωB97X-D/6-311G(d): the most significant ELF valence basin populations N for molecules **4a–h**, given as average number of electrons [e].

	4a	4b	4c	4d	4e	4f	4g	4h
ELF basins	N [e]	N [e]	N [e]	N [e]	N [e]	N [e]	N [e]	N [e]
V(C1,C2)	1.81	1.72	1.80	1.81	1.77	1.78	1.78	1.73
V(C1,C2)	1.75	1.83	1.75	1.74	1.77	1.78	1.76	1.81
V(C1,C_CN_)	2.39	2.39	2.39	2.39	2.39	2.39	2.39	2.39
V(C_CN_, N2)	2.54	2.56	2.57	2.60	2.60	2.60	2.62	2.63
V′(C_CN_, N2)	1.88	1.89	1.86	1.85	1.84	1.84	1.82	1.82
V(N2)	3.21	3.19	3.19	3.19	3.18	3.17	3.17	3.16
V(C1,N1)	2.30	2.28	2.25	2.29	2.29	2.29	2.25	2.26
V(N1,O1)	2.19	2.23	2.24	2.21	2.17	2.18	2.25	2.24
V(N1,O2)	1.84	1.84	1.85	1.84	1.85	1.86	1.85	1.86
V(O1)	2.89	2.85	2.83	2.84	2.81	2.85	2.8	2.83
V(O1)	2.77	2.8	2.82	2.81	2.83	2.80	2.84	2.80
V(O2)	2.82	2.78	2.81	2.79	2.80	2.79	2.80	2.79
V′(O2)	2.81	2.83	2.8	2.81	2.80	2.81	2.80	2.8
V(C6,R)	2.49	1.59	2.01		1.07	1.51	2.24	2.30

**Table 5:** ωB97X-D/6-311G(d): Global reactivity indices of **1a**–**h**. HOMO and LUMO energies as well as global reactivity given in electronvolts [eV].

	HOMO	LUMO	µ	η	ω	N	Electrophile	Nucleophile
**1a**	−8.11	1.48	−3.31	9.59	0.57	3.29	marginal	strong
**1b**	−7.26	1.84	−2.71	9.10	0.40	4.14	marginal	super
**1c**	−7.80	1.56	−3.12	9.36	0.52	3.59	marginal	strong
**1d**	−7.94	1.64	−3.15	9.58	0.52	3.46	marginal	strong
**1e**	−8.07	1.40	−3.34	9.47	0.59	3.32	marginal	strong
**1f**	−8.05	1.25	−3.40	9.29	0.62	3.35	moderate	strong
**1g**	−8.08	1.34	−3.37	9.42	0.60	3.32	moderate	strong
**1h**	−7.97	1.61	−3.18	9.59	0.53	3.42	marginal	strong

**Table 6:** ωB97X-D/6-311G(d): Global reactivity indices of **2a**–**h**, **3a**–**h**, **4a**–**h**. HOMO and LUMO energies as well as global reactivity given in electronvolts [eV].

	R	σ_p_	σ+	σ_R_	σ_I_	Homo	Lumo	µ	ŋ	ω	N	Electrophile	Nucleophile
**2a**	NH_2_	−0.66	−1.30	−0.76	0.10	−7.92	−0.58	−4.25	7.35	1.23	3.47	strong	strong
**2b**	OMe	−0.27	−0.78	−0.64	0.23	−8.37	−0.73	−4.55	7.64	1.36	3.02	strong	strong
**2c**	Me	−0.17	−0.31	−0.05	-0.08	−8.82	−0.84	−4.83	7.98	1.46	2.57	strong	moderate
**2d**	H	0.00	0.00	0.00	0.00	−9.09	−0.93	−5.01	8.16	1.54	2.31	strong	moderate
**2e**	F	0.06	−0.07	−0.44	0.50	−9.06	−1.00	−5.03	8.07	1.57	2.33	strong	moderate
**2f**	Cl	0.23	0.11	−0.36	0.51	−9.08	−1.13	−5.11	7.95	1.64	2.31	strong	moderate
**2g**	CF_3_	0.54	0.65			−9.56	−1.31	−5.44	8.25	1.79	1.84	strong	marginal
**2h**	NO_2_	0.78	0.79		0.63	−9.82	−1.77	−5.79	8.05	2.09	1.58	strong	marginal
**3a**	NH_2_	−0.66	−1.30	−0.76	0.10	−7.91	−0.72	−4.31	7.18	1.30	3.49	strong	strong
**3b**	OMe	−0.27	−0.78	−0.64	0.23	−8.34	−0.88	−4.61	7.46	1.42	3.06	strong	strong
**3c**	Me	−0.17	−0.31	−0.05	-0.08	−8.74	−0.96	−4.85	7.78	1.51	2.66	strong	moderate
**3d**	H	0.00	0.00	0.00	0.00	−8.95	−1.06	−5.01	7.88	1.59	2.45	strong	moderate
**3e**	F	0.06	−0.07	−0.44	0.50	−8.94	−1.13	−5.04	7.82	1.62	2.45	strong	moderate
**3f**	Cl	0.23	0.11	−0.36	0.51	−8.97	−1.25	−5.11	7.72	1.69	2.42	strong	moderate
**3g**	CF_3_	0.54	0.65			−9.42	−1.32	−5.37	8.10	1.78	1.97	strong	moderate
**3h**	NO_2_	0.78	0.79		0.63	−9.57	−1.84	−5.71	7.73	2.11	1.82	strong	marginal
**4a**	NH_2_	−0.66	−1.30	−0.76	0.10	−8.22	−1.18	−4.70	7.04	1.57	3.17	strong	strong
**4b**	OMe	−0.27	−0.78	−0.64	0.23	−8.72	−1.38	−5.05	7.34	1.74	2.68	strong	moderate
**4c**	Me	−0.17	−0.31	−0.05	-0.08	−9.17	−1.51	−5.34	7.67	1.86	2.22	strong	moderate
**4d**	H	0.00	0.00	0.00	0.00	−9.45	−1.60	−5.52	7.85	1.95	1.95	strong	moderate
**4e**	F	0.06	−0.07	−0.44	0.50	−9.41	−1.67	−5.54	7.75	1.98	1.98	strong	moderate
**4f**	Cl	0.23	0.11	−0.36	0.51	−9.40	−1.79	−5.60	7.61	2.06	1.99	strong	moderate
**4g**	CF_3_	0.54	0.65			−9.88	−1.96	−5.92	7.91	2.22	1.52	strong	marginal
**4h**	NO_2_	0.78	0.79		0.63	−10.12	−2.33	−6.23	7.79	2.49	1.27	super	marginal

**Table 7:** ωB97X-D/6-311G(d): The local electronic properties for **Cp** (**1a**–**h**).

		Parr P_k_-	N_k_	Parr P_k_-	N_k_	Parr P_k_-	N_k_	Parr P_k_-	N_k_	Parr P_k_-	N_k_
		C1		C2		C3		C4		C5	
**1a**	strong nucleophile	0.47	1.55	0.08	0.26	0.08	0.26	0.47	1.55	0.05	0.16
**1b**	super nucleophile	0.41	1.72	0.08	0.33	0.08	0.33	0.41	1.72	0.05	0.21
**1c**	strong nucleophile	0.39	1.41	0.16	0.58	0.03	0.11	0.47	1.67	0.05	0.18
**1d**	strong nucleophile	0.54	1.85	0.09	0.31	0.03	0.10	0.41	1.43	0.05	0.17
**1e**	strong nucleophile	0.46	1.54	0.08	0.27	0.08	0.27	0.46	1.54	0.05	0.17
**1f**	strong nucleophile	0.43	1.43	0.09	0.30	0.04	0.13	0.45	1.50	0.05	0.17
**1g**	strong nucleophile	0.50	1.64	0.09	0.30	0.04	0.13	0.44	1.45	0.05	0.17
**1h**	strong nucleophile	0.47	1.62	0.08	0.27	0.08	0.27	0.47	1.62	0.06	0.21

**Table 8:** ωB97X-D/6-311G(d): The local electronic properties for **CNA** (**2a**–**h**, **3a**–**h**, **4a**–**h**).

		Parr P_k_+	ω_k_	Parr P_k_+	ω_k_			Parr P_k_+	ω_k_	Parr P_k_+	ω_k_
		C1		C2				C1		C2	
**2a**	strong electrophile	0.07	0.08	0.31	0.38	**3e**	strong electrophile	0.08	0.13	0.32	0.52
**2b**	strong electrophile	0.08	0.11	0.30	0.40	**3f**	strong electrophile	0.11	0.18	0.28	0.48
**2c**	strong electrophile	0.10	0.15	0.27	0.40	**3g**	strong electrophile	0.11	0.19	0.29	0.52
**2d**	strong electrophile	0.10	0.16	0.27	0.42	**3h**	strong electrophile	0.15	0.31	0.15	0.32
**2e**	strong electrophile	0.10	0.16	0.28	0.44	**4a**	strong electrophile	0.02	0.04	0.47	0.74
**2f**	strong electrophile	0.13	0.21	0.24	0.40	**4b**	strong electrophile	0.04	0.07	0.46	0.80
**2g**	strong electrophile	0.14	0.26	0.21	0.38	**4c**	strong electrophile	0.05	0.10	0.44	0.82
**2h**	strong electrophile	0.16	0.34	0.10	0.21	**4d**	strong electrophile	0.06	0.12	0.44	0.85
**3a**	strong electrophile	0.04	0.06	0.35	0.45	**4e**	strong electrophile	0.06	0.11	0.44	0.88
**3b**	strong electrophile	0.06	0.09	0.33	0.47	**4f**	strong electrophile	0.07	0.15	0.41	0.85
**3c**	strong electrophile	0.08	0.12	0.32	0.49	**4g**	strong electrophile	0.09	0.20	0.39	0.86
**3d**	strong electrophile	0.08	0.13	0.31	0.49	**4h**	super electrophile	0.11	0.28	0.30	0.74

**Table 9:** Hammett correlation equations for the investigated series.

Reaction	Function Equation	R
**2**+**Cp**	Δω_2-**Cp**_ = 0.5698σ_p2_ + 1.1487+ω**_Cp_**	0.979
**3**+**Cp**	Δω_3-**Cp**_ = 0.5233σ_p3_ + 1.1941+ω**_Cp_**	0.970
**4**+**Cp**	Δω_4-**Cp**_ = 0.6156σ_p4_ + 1.5445+ω**_Cp_**	0.991

## Data Availability

The data that support the findings of this study are available from the corresponding author upon reasonable request.

## Supplementary Materials

The following supporting information can be downloaded at: https://www.mdpi.com/article/10.3390/molecules30112467/s1, Table S1. Global electrophilicity power ΔΩ of studied reactions. Table S2. Cartesian coordinated for the selected cyclopentadiene analogues and conjugated alkenes.
