# Supplementary material for: Modeling of the General Trends of Reactivity and Regioselectivity in Cyclopentadiene–Nitroalkene Diels–Alder Reactions
Source: Molecules. 2025 Jun 4;30(11):2467. doi: 10.3390/molecules30112467 (PMC12156356; doi:10.3390/molecules30112467)
Supplement: Supplementary file 1 [file molecules-30-02467-s001.zip › molecules-3653690-supplementary.pdf]

SUPPLEMENTARY MATERIAL

Modeling of the General Trends of Reactivity and Regioselectivity in Cyclopentadiene–Nitroalkene Diels–Alder Reactions

Adrianna Fałowska[1], Stanisław Grzybowski[1], Daniel Kapuściński[1], Karol Sambora[1], Agnieszka Łapczuk[1]*

[1] *Department of Organic Chemistry and Technology, Cracow University of Technology, Warszawska 24,31-155 Cracow, Poland*
* Correspondence: *agnieszka.lapczuk@pk.edu.pl*

Table 1. GLOBAL ELECTROPHILICITY POWER ΔΩ OF STUDIED REACTIONS.

		1a	1b	1c	1d	1e	1f	1g	1h
	ω (eV)	0.57	0.4	0.52	0.52	0.59	0.62	0.6	0.53
**2a**	1.23	0.66	0.83	0.71	0.71	0.64	0.61	0.63	0.7
**2b**	1.36	0.79	0.96	0.84	0.84	0.77	0.74	0.76	0.83
**2c**	1.46	0.89	1.06	0.94	0.94	0.87	0.84	0.86	0.93
**2d**	1.54	0.97	1.14	1.02	1.02	0.95	0.92	0.94	1.01
**2e**	1.57	1.0	1.17	1.05	1.05	0.98	0.95	0.97	1.04
**2f**	1.64	1.07	1.24	1.12	1.12	1.05	1.02	1.04	1.11
**2g**	1.79	1.22	1.39	1.27	1.27	1.2	1.17	1.19	1.26
**2h**	2.09	1.52	1.69	1.57	1.57	1.5	1.47	1.49	1.56
**3a**	1.3	0.73	0.9	0.78	0.78	0.71	0.68	0.7	0.77
**3b**	1.42	0.85	1.02	0.9	0.9	0.83	0.8	0.82	0.89
**3c**	1.51	0.94	1.11	0.99	0.99	0.92	0.89	0.91	0.98
**3d**	1.59	1.02	1.19	1.07	1.07	1.0	0.97	0.99	1.06
**3e**	1.62	1.05	1.22	1.1	1.1	1.03	1.0	1.02	1.09
**3f**	1.69	1.12	1.29	1.17	1.17	1.1	1.07	1.09	1.16
**3g**	1.78	1.21	1.38	1.26	1.26	1.19	1.16	1.18	1.25
**3h**	2.11	1.54	1.71	1.59	1.59	1.52	1.49	1.51	1.58
**4a**	1.57	1.0	1.17	1.05	1.05	0.98	0.95	0.97	1.04
**4b**	1.74	1.17	1.34	1.22	1.22	1.15	1.12	1.14	1.21
**4c**	1.86	1.29	1.46	1.34	1.34	1.27	1.24	1.26	1.33
**4d**	1.95	1.38	1.55	1.43	1.43	1.36	1.33	1.35	1.42
**4e**	1.98	1.41	1.58	1.46	1.46	1.39	1.36	1.38	1.45
**4f**	2.06	1.49	1.66	1.54	1.54	1.47	1.44	1.46	1.53
**4g**	2.22	1.65	1.82	1.7	1.7	1.63	1.6	1.62	1.69
**4h**	2.49	1.92	2.09	1.97	1.97	1.9	1.87	1.89	1.96

Table 2. CARTESIAN COORDINATES FOR THE SELECTED CYCLOPENTADIENE ANALOGUES AND CONJUGATED ALKENES.

1a

Center Number	Atomic Number	Atomic Type	Coordinates (Angstroms) X	Y	Z
1	6	0	1.530950	-0.693602	0.000197
2	1	0	2.073500	-1.066059	-0.878157
3	1	0	2.073156	-1.065844	0.878871
4	6	0	1.435540	0.803404	-0.000170
5	1	0	2.291249	1.464843	-0.000234
6	6	0	0.144222	1.160740	0.000056
7	1	0	-0.240009	2.174013	0.000126
8	6	0	0.094411	-1.122482	-0.000187
9	1	0	-0.228917	-2.155357	-0.000269
10	6	0	-0.704591	-0.042703	-0.000015
11	1	0	-1.774522	-0.054880	0.000058

1b

Center Number	Atomic Number	Atomic Type	Coordinates (Angstroms) X	Y	Z
1	6	0	-1.983122	0.590717	0.000000
2	1	0	-0.864947	0.590717	0.000000
3	6	0	-2.530184	-0.174922	-1.179921
4	6	0	-3.277652	-1.219872	-0.735867
5	6	0	-2.530263	-0.174899	1.179889
6	6	0	-3.277732	-1.219860	0.735909
7	6	0	-2.480560	2.048166	0.000000
8	1	0	-2.120007	2.548093	0.874628
9	1	0	-2.116801	2.549188	-0.872672
10	1	0	-3.550494	2.059858	-0.001956
11	6	0	-2.232076	0.241721	-2.632209
12	1	0	-2.225827	-0.626103	-3.258101
13	1	0	-2.987036	0.920199	-2.970739
14	1	0	-1.276237	0.720553	-2.676843
15	6	0	-4.029400	-2.271097	-1.573365
16	1	0	-4.727510	-1.780183	-2.218769
17	1	0	-3.328761	-2.825806	-2.161839
18	1	0	-4.554248	-2.937697	-0.921383
19	6	0	-4.029471	-2.270934	1.573604
20	1	0	-5.074446	-2.225949	1.347985
21	1	0	-3.655972	-3.246218	1.340755
22	1	0	-3.880306	-2.070925	2.614107
23	6	0	-2.232233	0.241670	2.632214
24	1	0	-3.102581	0.694623	3.059095
25	1	0	-1.963458	-0.622467	3.203116
26	1	0	-1.423586	0.942286	2.643514

1c

Center Number	Atomic Number	Atomic Type	Coordinates (Angstroms) X	Y	Z
1	6	0	0.193889	-1.165207	0.000196
2	1	0	0.006234	-1.802962	-0.877673
3	1	0	0.006408	-1.802695	0.878308
4	6	0	1.591337	-0.599779	-0.000207
5	1	0	2.490735	-1.204865	-0.000358
6	6	0	1.515542	0.746271	0.000044
7	1	0	2.352250	1.437792	0.000074
8	6	0	-0.692136	0.062955	-0.000079
9	6	0	0.105065	1.154859	0.000075
10	1	0	-0.235190	2.186274	0.000140
11	6	0	-2.188088	0.005634	-0.000077
12	1	0	-2.575382	-0.526334	-0.880465
13	1	0	-2.575373	-0.526109	0.880454
14	1	0	-2.623340	1.010507	-0.000196

1g

Center Number	Atomic Number	Atomic Type	Coordinates (Angstroms) X	Y	Z
1	6	0	1.530950	-0.693602	0.000197
2	1	0	2.073500	-1.066059	-0.878157
3	1	0	2.073156	-1.065844	0.878871
4	6	0	1.435540	0.803404	-0.000170
5	1	0	2.291249	1.464843	-0.000234
6	6	0	0.144222	1.160740	0.000056
7	1	0	-0.240009	2.174013	0.000126
8	6	0	0.094411	-1.122482	-0.000187
9	1	0	-0.228917	-2.155357	-0.000269
10	6	0	-0.704591	-0.042703	-0.000015
11	14	0	-2.643941	0.007517	0.000055
12	6	0	-3.266657	0.938564	1.584030
13	1	0	-3.034560	1.979714	1.500129
14	1	0	-4.325495	0.815538	1.676910
15	1	0	-2.783372	0.533957	2.448685
16	6	0	-3.266776	0.938386	-1.583978
17	1	0	-2.820052	0.502056	-2.452858
18	1	0	-4.331495	0.855792	-1.650688
19	1	0	-2.992303	1.970728	-1.522053
20	6	0	-3.337739	-1.804179	0.000183
21	1	0	-2.994841	-2.317357	0.874237
22	1	0	-4.407380	-1.776480	-0.000551
23	1	0	-2.993657	-2.317935	-0.873066

2a

Center Number	Atomic Number	Atomic Type	Coordinates (Angstroms) X	Y	Z
1	6	0	-2.711392	-0.636313	0.190336
2	6	0	-1.834231	-0.210692	-0.816320
3	6	0	-1.491659	1.144198	-0.920427
4	6	0	-2.026249	2.073468	-0.017879
5	6	0	-2.903413	1.647847	0.988776
6	6	0	-3.245984	0.292956	1.092883
7	1	0	-1.426058	-0.920210	-1.505435
8	1	0	-0.821924	1.469169	-1.689029
9	1	0	-3.311584	2.357365	1.677892
10	1	0	-3.915717	-0.032015	1.861487
11	6	0	-1.649793	3.562358	-0.132279
12	6	0	-0.801546	3.973947	-1.105747
13	1	0	-2.057963	4.271875	0.556838
14	1	0	-0.393377	3.264429	-1.794865
15	7	0	-0.442198	5.395159	-1.214945
16	8	0	-0.914302	6.215812	-0.417887
17	8	0	0.332441	5.771032	-2.103937
18	7	0	-3.070731	-2.057528	0.299542
19	1	0	-3.261354	-2.280650	1.255512
20	1	0	-3.883707	-2.239260	-0.253670

3a

Center Number	Atomic Number	Atomic Type	Coordinates (Angstroms) X	Y	Z
1	6	0	0.273506	-4.024575	-0.172234
2	6	0	1.234430	-3.818885	0.826884
3	6	0	2.039889	-2.672420	0.799019
4	6	0	1.884424	-1.731646	-0.227965
5	6	0	0.923503	-1.937338	-1.227086
6	6	0	0.118043	-3.083803	-1.199221
7	1	0	1.353131	-4.537187	1.611009
8	1	0	2.773575	-2.515370	1.561869
9	1	0	0.804804	-1.219038	-2.011213
10	1	0	-0.615642	-3.240853	-1.962071
11	6	0	2.769541	-0.471793	-0.258585
12	1	0	3.503229	-0.314744	0.504264
13	6	0	2.619201	0.437967	-1.251712
14	35	0	1.309539	0.157624	-2.613436
15	7	0	-0.571384	-5.227159	-0.143003
16	1	0	-0.706506	-5.516650	0.804593
17	1	0	-0.124832	-5.960796	-0.655221
18	7	0	3.464087	1.640553	-1.280941
19	8	0	4.312695	1.822202	-0.398601
20	8	0	3.326792	2.471368	-2.187886

4a

Center Number	Atomic Number	Atomic Type	Coordinates (Angstroms) X	Y	Z
1	6	0	-1.391435	0.053892	-0.511533
2	6	0	-0.688737	0.260336	0.683255
3	6	0	-1.131768	-0.340160	1.869446
4	6	0	-2.277496	-1.147102	1.860849
5	6	0	-2.980194	-1.353545	0.666062
6	1	0	-1.053169	0.512382	-1.417217
7	1	0	0.186053	0.876453	0.689819
8	1	0	-2.615760	-1.605594	2.766533
9	1	0	-3.854985	-1.969661	0.659498
10	6	0	-2.537163	-0.753050	-0.520130
11	6	0	-3.309358	-0.979912	-1.833084
12	6	0	-4.417315	-1.760251	-1.841397
13	1	0	-2.971095	-0.521418	-2.738767
14	6	0	-4.904161	-2.420139	-0.537891
15	7	0	-5.266643	-2.911456	0.432627
16	7	0	-5.154409	-1.976803	-3.094672
17	8	0	-4.763163	-1.446493	-4.142217
18	8	0	-6.166221	-2.689428	-3.102264
19	7	0	-0.394672	-0.123609	3.122721
20	1	0	0.573628	0.021193	2.919185
21	1	0	-0.760991	0.680971	3.590120
